# A Study on the Borehole Wall Stability Analysis and Slurry Ratio Optimization for Construction of Pile in Complex Marine Strata

**DOI:** 10.3390/ma17091984

**Published:** 2024-04-24

**Authors:** Qingxiong Zhao, Linglin Xie, Ping Cao, Ziyang Zhang, Kaihui Li, Hang Lin, Chao Huang

**Affiliations:** 1 School of Resources and Safety Engineering, Central South University, Changsha 410083, China; qingxiongzhao@csu.edu.cn (Q.Z.); pcao_csu@csu.edu.cn (P.C.); 195501002@csu.edu.cn (Z.Z.); geokhli@csu.edu.cn (K.L.); 2 Key Laboratory of Natural Resources Monitoring and Supervision in Southern Hilly Region, Ministry of Natural Resources, Changsha 430071, China; 3 The Second Surveying and Mapping Institude of Hunan Province, Changsha 430071, China; 4 Sinohydro Engineering Bureau 8 Co., Ltd., Changsha 410004, China; 185511012@csu.edu.cn

**Keywords:** bored cast-in-place piles, discrete element modelling, mud-wall-protection effect, mud ratio optimization, mud slurry viscosity, type of additions in mud slurry

## Abstract

In order to address the issue of hole collapse, which frequently arises when boring piles are being constructed in intricate marine strata, this paper discusses the influence of the slurry ratio on the slurry performance as well as the mechanism of slurry wall protection. It performs this by means of theoretical analysis, laboratory ratio testing, engineering analogies, numerical simulation, and field testing. Our findings demonstrate that adding sodium polyacrylate and sodium carboxymethyl cellulose can enhance mud’s viscosity, contribute to flocculation, and improve the connection between mud and soil layers. Refering similar engineering cases, three optimization schemes are proposed for achieving a mud ratio that offers wall protection in complex marine strata. Furthermore, the particle flow model of slurry viscous fluid is established. The collapse of holes in the sand layer is reflected in the uneven radial displacement of hole walls and the invasion of mud particles. Increasing the viscosity of mud gradually transforms the uneven radial deformation of pore walls in the sand layer into a uniform radial deformation, whereas increasing the proportion of mud significantly decreases the radial displacement of hole walls. Additionally, when the mud pressure in the hole is 300 kPa and 600 kPa, the wall protection effect is better, and there is no particle penetration by substances such as sand. It is found that a high mud pressure can promote the diffusion of mud particles into the sand layer, while low mud pressure cannot balance the pressure on deep soil. The results of the field tests show that the ratio of water–clay–bentonite–CMC-Na–sodium carbonate = 700:110:90:1.5:0.5 used (where the mass percentage of each material is 77.8% water, 12.2% clay, 10% bentonite, 0.16% CMC-Na, and 0.05% sodium carbonate) can effectively prevent hole collapse and reduce the thickness of the sand layer at the bottom of the hole by 50%.

## 1. Introduction

Mud slurry drilling piles have gained significant popularity in contemporary engineering projects due to their versatility across different strata, effective wall protection capabilities, and straightforward construction techniques [1]. Nevertheless, the construction of foundations in marine facies strata presents numerous challenges, including the traversal of multiple strata and intricate geological conditions, which often result in necking and collapse. These issues frequently lead to construction delays and substantial economic losses, particularly in complex marine facies strata [2]. Consequently, ensuring the stability of borehole walls has emerged as a prevalent goal in the field of foundation engineering [3].

Numerous engineering examples have shown that the stability of drilled-shaft walls is influenced by various factors, with mud slurry protection playing a vital role [4]. Scholars have conducted theoretical analysis [5,6], laboratory experiments [7,8,9,10,11], field experiments [12,13,14], and numerical simulations [15,16,17,18] to study the issue of mud slurry protection and soil stability. The unreinforced and geogrid-reinforced soil foundations were modeled using a particle flow code of two dimensions (PFC2D) and the effects of four different factors on footing settlement were investigated [19]. However, research on the micro–macro mechanism and characteristics of the mud slurry protection process is still lacking. Although numerical simulation methods, particularly finite element methods, have proved effective in studying the micro–macro mechanism of mud slurry, they face limitations in simulating the interaction between discrete media at the micro–macro scale. Discrete element methods, on the other hand, offer advantages in terms of simulating the interaction between mud slurry and soil in mud slurry–soil interactions. For example, discrete element methods have been used to study the stability of horizontal wells in a way that considers fractures [20], simulate fluid flow and rock-consolidation grouting technology [21], and study the diffusion patterns of slurry in sand grouting [22].

The composition and ratio of mud slurry materials significantly impact the performance characteristics of mud slurry. Extensive research has been conducted on mud slurry mix ratios and ingredients, leading to valuable results [23,24,25,26,27]. For instance, studies have compared the performance and wall protection effects of mud slurries with different soda ash and carboxymethyl cellulose sodium concentrations [24]. Researchers have also synthesized high-viscosity, high-stability, and collapse-resistant slurries using plant gum, sulfonated copolymers, and plant fiber powder [28]. Furthermore, in geologically complex areas, it is often necessary to configure mud slurries that are suitable for certain strata. Researchers have developed new polymer slurry formulations that are suitable for specific projects, such as the Shanghai Fuxing East Road Yangtze Tunnel project [29] and the Beijing Urban Sub-center Tongzhou District Lucheng Town comprehensive gallery project [30].

To date, there have been many studies on the stability of the hole walls of bored piles under the action of mud walls. Previous research has explored the mechanical model of hole-wall instability, mud ratios, and drilling construction technology, obtaining fruitful research results. However, the mechanism of mud action, the applicability of mud in different strata, and the stability mechanism of hole wall in complex strata remain to be studied. Moreover, the existing theories about mud ratios and hole-wall stability are still limited to individual engineering examples, which are not necessarily applicable to engineering in complex geological conditions.

To address this gap, the stability coefficient of the geological strata, derived through deduction, is employed to analyze engineering cases and propose solutions for borehole stability management, with a focus on the pile foundation of the Xiaomeisha seaside tourist area. Further indoor experiments are conducted to explore the effects of solid-phase composition, the solid–liquid ratio, and slurry additives on slurry performance. Combined with the similar engineering experience, the optimal slurry ratio scheme for marine sand-layer construction is given. Additionally, the numerical model of viscous slurry fluid is established based on the discrete element method used to investigate its fluid and viscosity properties. Finally, the particle-based model of borehole walls in complex strata is established to explore the influences of slurry performance and slurry pressure and evaluate the stability of borehole walls.

## 2. Project Overview

**Figure 1:**
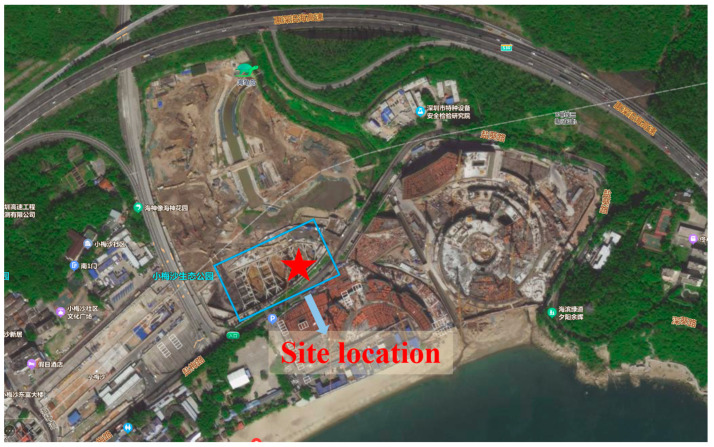
Site plan (source: Baidu Satellite Map).

The project, referred to as Area 03-01-2, is situated in the southwest area of Meisha Beach Tourism District, Meisha Street, Shenzhen City, Guangdong Province, China. Its location and surrounding environment are shown in Figure 1. It is designated for the development of recreational facilities. The scope of the project entails the construction of a two-story underground basement (partially three-story), with an excavation area of 8350 m^2^ and a support perimeter of 412 m. Taking into consideration the thickness of the bottom plate and cushion layer, the excavation depth will range from approximately 9.5 m to 15.0 m. Additionally, a secondary excavation pit with a depth of 6.0 m and a support length of 144 m will be incorporated into the project.

### 2.1. Hydrogeological and Geological Engineering Conditions

The site is situated approximately 200 m south of Xiaomeisha Bay, with the Xiao meisha River and Shenzhen Deep Pit Water bordering the northeastern side of the site. On the northern side of the project site, there is an artificial lake. The main types of groundwater present at the site include fourth-period pore water and bedrock-fissure water. Based on on-site drilling, in situ testing, and laboratory soil tests, the following strata were identified at the site: artificial filling soil (Qml), a Holocene-series intertidal and estuarine sediment layer (Q4mc), an Upper Quaternary-series alluvial and flood deposit layer (Q3al + pl), a residual layer of the Quaternary series (Qel), and an underlying bedrock composed of Late Mesozoic Yanshanian Stage V coarse-grained granite (γK2). The hydrogeological conditions at the site were referenced from the text report of Area 03-01-2 provided by the Shenzhen Investigation & Research Institute Co., Ltd. (Shenzhen, China). The recommended values of rock–soil parameters for the design of the excavation pit’s support are listed in Table 1. The soil state fundamentally shapes a pit’s behavior and engineering capabilities, being particularly significant in the design and construction processes, and encompasses various states such as ‘loose’ and ‘elastoplastic’. The rapid-consolidation shear test is a vital tool for evaluating the shear strength of saturated soils. It entails a three-axis test that yields the cohesion (c) and internal friction angle (φ) of the soil, as well as the ultimate bond strength between the rock/soil layer and the anchor grouting (in kPa). These findings are vital for understanding bonding properties. On-site grouting tests refine these bond strength parameters, offering a more precise measure of bond strength. The friction coefficient (μ) between the rock/soil and the retaining wall substrate is a pivotal factor in the assessment of slip resistance. This coefficient can be measured directly in the field using a flat-shovel test (DMT), enabling a more accurate assessment of the friction characteristics between the substrate and the rock/soil layer.

### 2.2. Excavation-Pit Drilling Pile Construction Plan

The excavation-pit support plan for this project is as follows. The diameter of the drilling piles will be 1200 mm with a spacing of 1800 mm, and a one-layer reinforced-concrete support will be installed vertically. Triaxial cement–soil mixing piles will be installed between the piles. For the inner pit, sheet piles with a diameter of 1000 mm and a spacing of 1200 mm will be used, with a one-layer, reinforced concrete support installed vertically.

The drilling and excavation process encompasses a series of intricate steps, including measurement and payoff, mud preparation, the installation of buried casing, the operation of drilling rigs, hole inspection, sediment removal at the bottom of the hole, the installation of a reinforcement cage, and the subsequent lowering of the conduit in order to pour concrete. Throughout this process, the drilling phase is particularly critical. Insufficient support from the hole wall or construction disturbances can lead to the collapse of the wall into the hole. This collapse not only presents challenges in continuing the hole-cleaning process, but also compromises the integrity of the surrounding hole wall, potentially leading to further collapse.

This project is situated in a complex marine facies stratum, characterized by several meters of marine gravel–sand layers, a shallow groundwater level, long pile length, and large pile diameter. The instability of the marine sandy strata leads to phenomena such as wall collapse during drilling and borehole collapse during backfilling.

## 3. Causes of Borehole Wall Collapse and Analysis of Wall Stability

**Figure 2:**
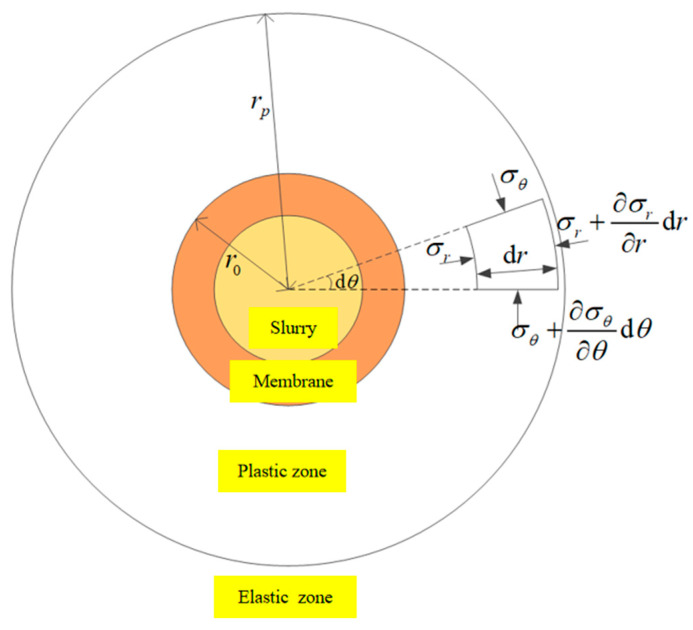
Calculation area division.

The drilling of piled foundations in the presence of a thick marine sand layer causes a high risk of borehole wall collapse due to the poor stability of the sand layer when exposed to water. The pressure difference between the inside and outside of the borehole wall is the main factor contributing to wall collapse. The mud film formed by the infiltration of mud slurry into the soil acts as a transitional zone between the mud slurry and the soil, effectively balancing the pressure difference at the borehole wall. Considering the stability of the hole wall under the action of mud, the calculation area is divided as shown in Figure 2 [31,32]. Due to the process of borehole excavation and the action of drilling mud, the surrounding rock is segmented into four distinct zones: an outermost elastic zone, a subsequent plastic zone, a membrane zone, and a zone filled with slurry. At point r from the borehole’s center, a unit of surrounding rock exists, within which the hoop stress and radial stress of the rock unit are, respectively, $\sigma_{r},\sigma_{\theta }$.

The force exerted by the slurry on the mud film can be expressed as show in Equation (1):

(1) $$\sigma_{x}=\gamma_{m}y$$

According to Bishop’s solution [33], in a borehole wall with a pile diameter of *d*, the horizontal stress exerted by the slurry on the mud film is given by the following Equation (2):

(2) $$\sigma_{x}=2\tau_{m}y/d+\pi \tau_{m}/2$$

Thus, the force exerted by the mud film on the borehole wall is expressed by the following Equation (3):

(3) $$P_{m}=\int_{0}^{H}\sigma_{x}dy=\tau_{m}H^{2}/d+\frac{1}{2}\pi \tau_{m}H$$

To ensure borehole wall stability in the marine sandy strata, the following condition, expressed in Equation (4), must be satisfied:

(4) $$\left\{\begin{array}{c} \gamma_{m}y\geq \frac{k\gamma y}{(\frac{r_{0}}{r_{p}}{)}^{\frac{2\sin\varphi }{1-\sin\varphi }}}\\ 2\tau_{m}y/d+\pi \tau_{m}/2\geq \gamma_{m}y-\frac{k\gamma y}{(\frac{r_{0}}{r_{p}}{)}^{\frac{2\sin\varphi }{1-\sin\varphi }}} \end{array}\right.$$

The description of borehole wall stability using safety factors can be divided into slurry-stability safety factors (characterizing the stability of slurry diffusion into surrounding rock soil) and mud-film-stability safety factors (characterizing the possibility of preventing surrounding rock soil from collapsing), represented by the following Equation (5):

(5) $$\left\{\begin{array}{c} F_{s1}=\frac{\gamma_{m}y(\frac{r_{0}}{r_{p}}{)}^{\frac{2\sin\varphi }{1-\sin\varphi }}}{k\gamma y}\\ F_{s2}=\frac{2\tau_{m}y/d+\pi \tau_{m}/2}{\gamma_{m}y-\frac{k\gamma y}{(\frac{r_{0}}{r_{p}}{)}^{\frac{2\sin\varphi }{1-\sin\varphi }}}} \end{array}\right.$$

According to the theory of circular hole expansion, the plastic zone radius $r_{p}$ is calculated using the following Equation (6):

(6) $$r_{p}=r_{0}{\left(1-\sin\varphi \right)}^{\frac{1-\sin\varphi }{2-\sin\varphi }}$$

where $F_{s1}$ represents the slurry-stability safety factor; $F_{s2}$ represents the mud-film-stability safety factor; $\tau_{m}$ represents the shear strength of the mud film; d represents the diameter of the drilled pile; H represents the borehole depth; $r_{0}$ represents the radius of the borehole; $r_{p}$ represents the plastic zone radius of the soil; γ represents the weight of the soil; y represents the depth of the soil; and k represents the lateral pressure coefficient of the soil.

Based on the equations presented, several observations can be made. Firstly, an increase in mud slurry density, a decrease in soil weight, and an increase in the internal friction angle of the sandy soil increase the slurry’s stability safety factor. Additionally, an increase in the shear strength of the mud film, a decrease in borehole diameter, and a decrease in borehole depth increase the mud-film-stability safety factor, facilitating the formation of a stable mud film. Sandy soil, compared to clay, has a larger internal friction angle but lacks cohesion, resulting in a lower shear strength of the mud film. The stability of the mud film is crucial in the stability of borehole walls in sandy soil layers, with the slurry stability being less significant. The instability of the mud film in sandy soil layers often leads to the widespread infiltration of mud slurry, weakening the soil strength and causing collapse. This aligns with the findings of Ma et al. [33] based on field examples. These equations can partially account for borehole stability in complex geological conditions.

Accordingly, in order to enhance the slurry-stability and mud-film-stability safety factors after determining soil and pile parameters, it is essential to optimize the materials and mix ratios of the wall protection mud slurry in order to reinforce the borehole wall and prevent collapse. The article “Technical Standard for Drilled Pile Construction” [34] primarily focuses on the performance requirements of slurry in general strata and lacks applicability. It also does not consider the application of additives. Further research is imperative to understanding the characteristics and performance of mud slurries for drilled piles in different strata.

## 4. Optimization of Mud Slurry Mix Ratio and Performance Testing Experiment

In order to optimize the mud slurry mix ratios of drilling piles in marine sandy strata, laboratory experiments were conducted to investigate the influence of different components and additives on the mud slurry’s characteristics, with the aim of developing new additives to improve the slurry’s performance.

### 4.1. Experimental Design

#### 4.1.1. Experimental Materials

In permeable sand and gravel strata where a bentonite–polymer slurry fails to form a mud film on the surface and instead directly infiltrates into the soil, the effective supporting pressure of the slurry significantly decreases, rendering it incapable of maintaining the borehole wall stability and leading to potential collapse. On the other hand, compared to other polymer slurries, the clay–polymer mud slurry has a lower viscosity and a higher density, making it unsuitable for the formation of mud slurry walls in marine layers [4]. To overcome the limitations of both types of slurry, it is necessary to harness their respective strengths. Hence, a mixed solid-phase polymer slurry, consisting of clay and bentonite as the solid-phase material, was selected for wall protection in drilling piles in the marine sandy strata. Furthermore, to optimize the performance of the mud slurry and enhance its effectiveness in terms of wall protection, variations in the mixtures of solid-phase materials and the effects of different additives and their concentrations on mud slurry characteristics (density, viscosity, sand content, and pH) were studied, analyzing their individual advantages and disadvantages.

**Figure 3:**
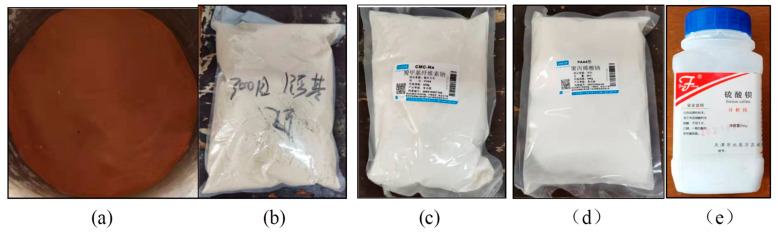
Experimental materials. (**a**) Clay; (**b**) bentonite; (**c**) CMC-Na; (**d**) sodium polyacrylate; (**e**) barium sulfate.

Figure 3 shows the experimental materials employed, including clay, bentonite, carboxymethyl cellulose sodium (CMC-Na), sodium polyacrylate, and barium sulfate powder. The clay utilized in the test was Changsha laterite, which is an acidic soil with low organic matter that is commonly found in hills and hills. Clay particles of less than 250 μm, which are easily slurryed, were screened and dried as test materials. Na-bentonite was chosen as bentonite, while montmorillonite was the primary hydrous clay mineral. It possessed the properties of swelling, adhesion, adsorption, catalysis, thixotropy, suspension, and cation exchange. The sodium bentonite colloidal suspension exhibited good thixotropy, viscosity, lubricity, a high PH value, thermal stability, plasticity, and strong adhesion.

#### 4.1.2. Experimental Equipment

**Figure 4:**
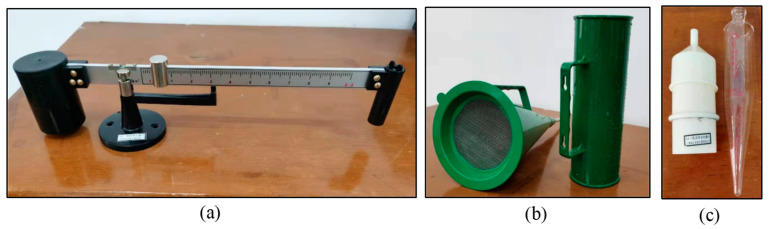
Experimental equipment adopted in this study: (**a**) NB-1 mud density meter; (**b**) 1006 Mud Viscosity Meter; (**c**) NA-1 Mud Sand Content Meter.

The equipment used to measure the performance parameters of the mud slurry and the corresponding details are shown in Figure 4 and Table 2, respectively. The NB-1 mud density meter was used to gauge the density of the mud slurry during the experiment. With an unequal arm, the NB-1 mud gravimeter is a balance. Its knife-lever edge is located on the seat that is fastened to the workbench using adhesive. The calibrated code device on the left side of the lever enables the moving code to read the mud weight on the ruler instantly. The viscosity of the mud slurry was measured using the 1006 Mud Viscosity Meter (Equipmentimes, Dalian, China), and the viscosity of the mud was calculated by measuring the number of seconds required for 500 cubic centimeters of muck to pass through the 1006 Field Standard Viscometer. The NA-1 Mud Sand Content Meter was employed to assess the sand content within the mud slurry, calculating the sand content per unit volume, subsequent to particle separation, flow rate measurement, and settlement analysis. pH test paper was utilized to determine the pH value of the mud slurry. Additionally, a precision electronic balance was used for material weighing, a microstirrer was employed for mud slurry mixing, and measuring cylinders and beakers were utilized to measure liquid volumes. 

To examine the impact of varying quantities of clay, bentonite mud slurry, water, CMC-Na, sodium polyacrylate, barium sulfate, among other factors, on the mud slurry characteristics and to assess the influence of different proportions of mud slurry components on its performance, a controlled-variable approach was employed. The types and quantities of slurry additives were maintained at constant levels, while the amounts of different solid-phase materials in the slurry were systematically adjusted to investigate their effects on mud slurry characteristics. Comparative analysis was conducted, and an optimal ratio was determined based on the specific requirements of the project.

### 4.2. Results of Mud Slurry Mix Ratio Testing and Material Characteristics Analysis

A comprehensive examination of the effects of various components and quantities of slurry, water, CMC-Na, sodium polyacrylate, bentonite, and clay on mud slurry characteristics was conducted. The corresponding mud slurry density, viscosity, sand content, and pH values are listed in Table 3, Table 4 and Table 5. These tables provide an analysis of the performance of the mud slurry under different component proportions.

#### 4.2.1. Effects of Clay and Bentonite Content

**Figure 5:**
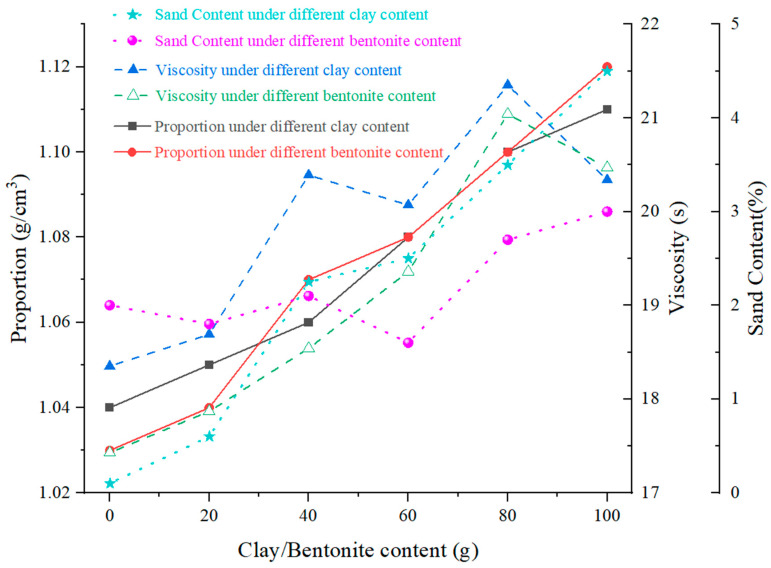
Effects of clay and bentonite content on mud slurry characteristics.

The effects of clay and bentonite content on mud slurry characteristics are illustrated in Figure 5. It can be observed that, as the content of clay and bentonite increases, the mud slurry density, viscosity, and sand content all exhibit upward trends. Moreover, both clay and bentonite contribute similarly to the increase in mud slurry density. By adding 0 to100 g of clay or bentonite, the density of the mud slurry can be enhanced from approximately 1.03 to 1.11. Overall, elevating the solid-phase content, whether it is clay or bentonite, significantly raises the mud slurry density. In terms of viscosity, a small amount of clay or bentonite, when combined with a certain quantity of additives, helps to maintain a viscosity of around 18 s, which is slightly higher than that of water. As the content of clay and bentonite is further increased, adding 80 to 100 g of clay or bentonite can increase the viscosity to around 20 s, with the clay slurry demonstrating a slightly higher viscosity than the bentonite slurry. When the amount of clay in the mud increases, it is possible that all of the CMC-Na molecules become fully encased in clay particles, creating a stable gel structure that will raise the mud’s viscosity even more. However, if there is an excessive amount of clay present, CMC-Na molecules can become overfilled in the spaces between the clay particles. This would prevent the CMC-Na molecules from combining with water in an efficient manner, which would lower the slurry’s viscosity [35]. However, the impact of solid-phase content on the viscosity enhancement is relatively minor. Regarding the sand content, the sieving process during the preparation of bentonite can ensure that particle size is generally smaller, resulting in the sand content of mud slurry remaining around 2%. Conversely, clay possesses larger particles. As the content of clay increases from 0 to 100 g, the sand content of the mud slurry rises from 0.1% to 4.5%, but the overall sand content still meets the required specifications [35]. Considering that subsequent additives can adjust viscosity, the contributions of clay and bentonite to the mud slurry density are comparable. From an engineering and economic perspective, it is suggested that the clay content should be slightly higher than the bentonite content. Accordingly, an optimal clay-to-bentonite ratio of 11:9 is selected for the subsequent experiments.

#### 4.2.2. Influence of Water Content

**Figure 6:**
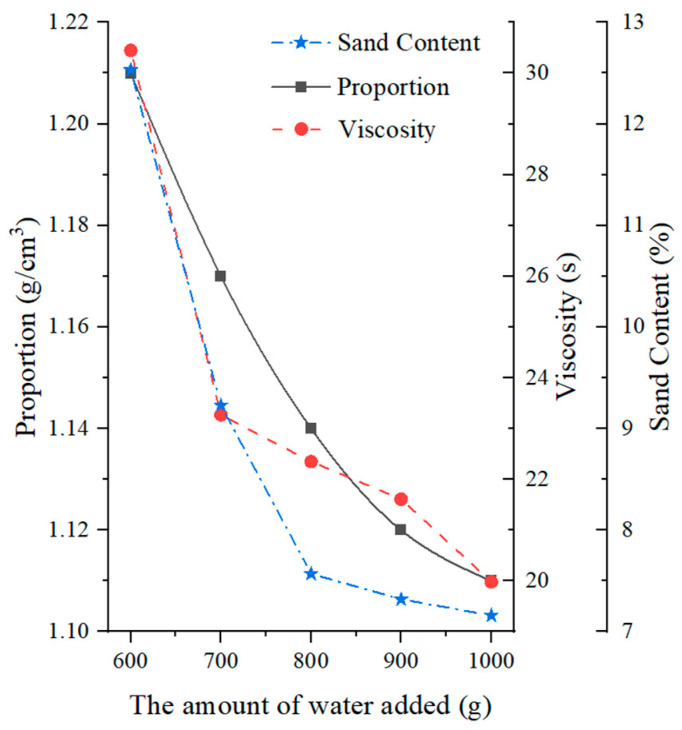
Effects of water content on mud slurry characteristics.

The impact of water content on slurry characteristics is illustrated in Figure 6. As the water content increases, the specific gravity, viscosity, and sand content of the slurry all decrease. More specifically, the specific gravity of the slurry decreases from 1.21 at 600 mL of water to 1.11 at 1000 mL of water, indicating the significant influence of water on specific gravity. The viscosity of the slurry reaches its maximum value of 30.45 s at 600 mL of water and then decreases significantly and stabilizes as the water content increases. Generally, the viscosity can be maintained above 20 s. The sand content also reaches its maximum of 12.53% at 600 mL of water and then decreases significantly with increasing water content, reaching a stable state above 800 mL of water. Accordingly, in order to reduce the sand content, it is recommended to have a higher water content in the slurry. The specific formulation used in this experiment was determined to be 1000 mL of water, 110 g of clay, and 90 g of bentonite.

#### 4.2.3. Influence of Slurry Additives

**Figure 7:**
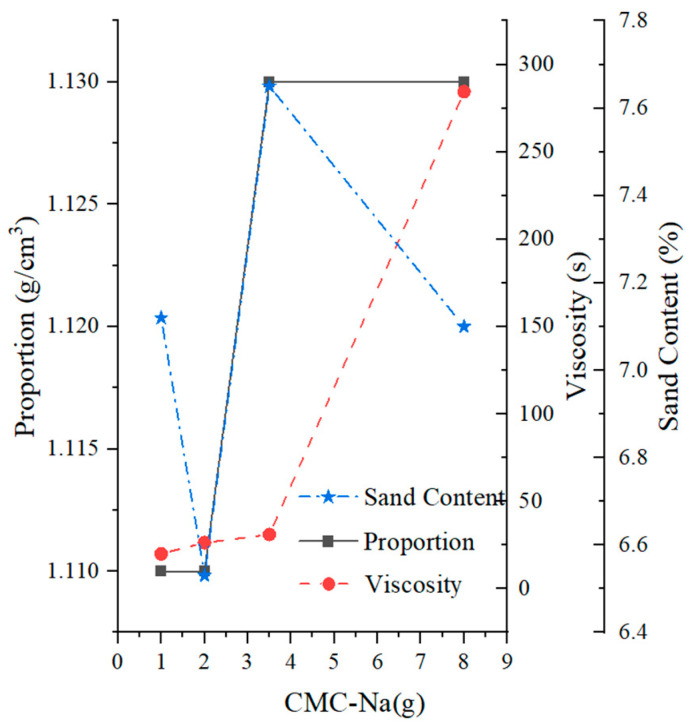
Effects of CMC-Na on mud slurry characteristics.

The influence of CMC-Na on slurry characteristics can be observed in Figure 7. The addition of CMC-Na had a minimal impact on the specific gravity and sand content of the slurry, which remained around 1.1 g/cm^3^ and 7%, respectively. In contrast, CMC-Na had a significant effect on the viscosity of the slurry. The viscosity increased significantly within the range of 1–3.5 g of CMC-Na, reaching 20–30 s, which met the common requirements. Beyond 3.5 g, the viscosity rapidly increased, reaching 284.44 s at 8 g. At this point, the viscosity exceeded the practical requirements of engineering [36].

**Figure 8:**
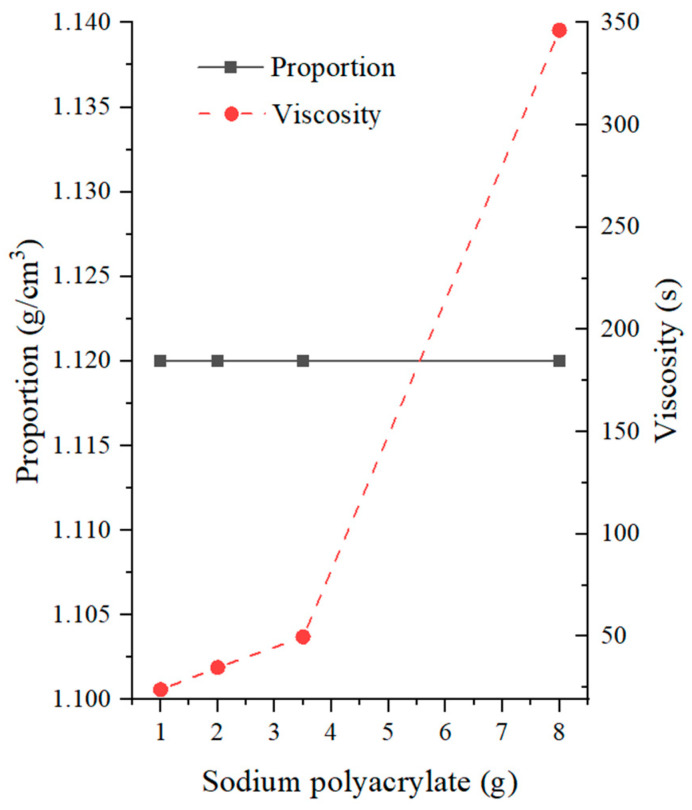
Effects of sodium polyacrylate on mud slurry characteristics.

The experimental results on the effects of sodium polyacrylate content on slurry characteristics are presented in Figure 8. Sodium polyacrylate had little effect on the specific gravity of the slurry, with overall specific gravity being maintained at 1.12 g/cm^3^. However, compared to CMC-Na, sodium polyacrylate had a more significant impact on the viscosity of the slurry. Within the range of 1–2 g, the viscosity of the slurry increased significantly and could reach 20–35 s, meeting the common requirements. Above 3.5 g, the viscosity of the slurry increased rapidly, reaching 346.25 s at 8 g. At this point, the viscosity became too high and did not meet the engineering requirements. In addition, sodium polyacrylate acted as a flocculant in the slurry [37], enhancing the dispersion of solid particles in the water and effectively reducing the slurry loss. However, it also made the solid particles in the slurry more viscous and coarser, significantly increasing the sand content of the slurry. At 1 g, 30% of the flocculation structure in the slurry could not pass through a 200-mesh sieve. Therefore, when the content was greater than or equal to 1.5, the sediment’s concentration was not obtained.

**Figure 9:**
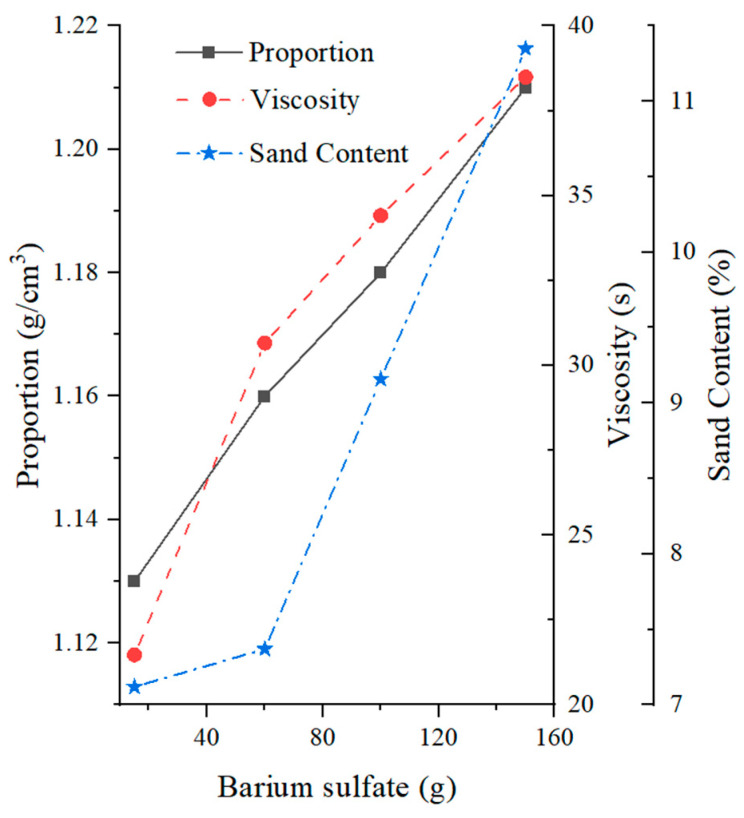
Effects of barium sulfate on mud slurry characteristics.

The experimental results regarding the effects of barium sulphate content on slurry characteristics are depicted in Figure 9. Barium sulphate itself is insoluble in water and is commonly used as a weighting agent in slurry preparation. The results show that, with the addition of large amounts of barium sulphate, the specific gravity and viscosity of the slurry increase significantly. However, considering that barium sulphate acts as a solid particle and increases the content of solid particles in the slurry at higher concentrations, the impact on specific gravity and viscosity is limited. The increase in specific gravity and viscosity is primarily due to the increase in solid particle content. Furthermore, the barium sulphate has larger particles, and its effect on the sand content in the slurry is minimal at lower doses, but significantly increases the sand content when the dose exceeds 60 g.

### 4.3. Optimization of Slurry Proportioning

#### 4.3.1. Analysis of the Specific Gravity Parameters of Slurry

**Figure 10:**
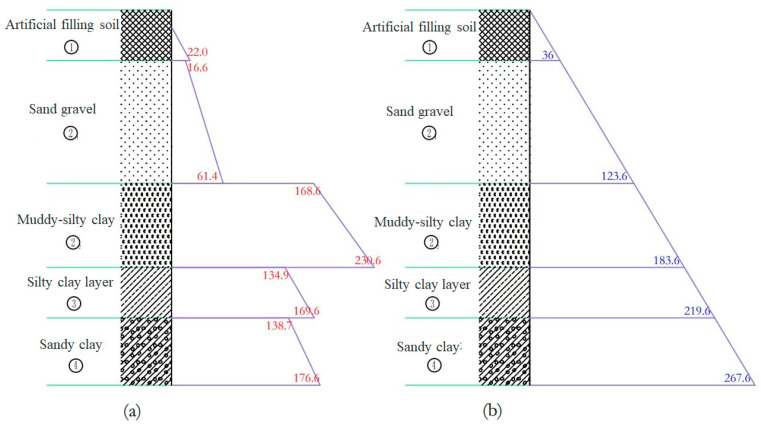
Distribution of active earth pressure of soil layers and liquid static pressure of slurry: (**a**) active earth pressure of soil layers and (**b**) liquid static pressure of slurry.

Based on engineering geology and hydrogeology, the Rankine active earth pressure theory is used to calculate the formation pressure distribution, as shown in Figure 10a. When the slurry performs well and forms a high-quality slurry skin, it acts as effective liquid pressure on the pore wall. The liquid static pressure of the slurry with a specific gravity of 1.2 is displayed in Figure 10b. However, it should be noted that the calculated soil pressure obtained using Rankine’s theory shows the action of retaining walls on the soil to be horizontal, while the actual excavation stress condition has a better arch effect than the retaining wall. Therefore, the calculated soil pressure tends to be overestimated. Additionally, sand may mix with the slurry during use, which increases its specific gravity. Consequently, the actual required specific gravity of the slurry should be less than 1.2. To avoid excessive levels of specific gravity, leading to water seepage into the soil layer and difficulty in drilling, it is considered to be more suitable to have a specific gravity of 1.14–1.18 during slurry formulation.

#### 4.3.2. Analysis of Slurry Viscosity Parameters

In low-permeability clay and clayey soil formations, where the pore size is small, the slurry has a tendency to form a clay film on the excavation surface. However, in highly permeable formations such as fine sand and gravel, the slurry easily penetrates and fails to form a clay film, resulting in a significant slurry loss and the inability to maintain slurry pressure.

Previous research [38] has shown that slurry properties have a substantial impact on the quality of clay films. Higher slurry viscosity, under the same degrees of gradation and density, leads to better physical stability, increased compactness, stronger bonding between slurry particles and water, and the clogging of pores in the formation by slurry particles during film formation. This makes it challenging for water in the slurry to penetrate into the formation, resulting in reduced filtrate.

In this project, the gravel layer is thick, with a maximum depth of 7.3 m, making it susceptible to collapse. Accordingly, it is necessary to control the viscosity of the slurry to ensure the quality of the clay film. Referring to viscosity values from engineering examples listed in Table 6, the slurry viscosity is determined to be 25–30 s using the engineering analogy method.

#### 4.3.3. Optimization of Slurry Proportioning

The originally planned slurry for the project was a bentonite slurry, prepared with a water-to-bentonite ratio of 1000:200. After sufficient stirring and standing for 3 h, the slurry reached a gel state. By selecting one of the precipitation wells as a test pile, the specific gravity of the original slurry was determined to be 1.11 g/cm^3^ and the viscosity was found to be 20.14 s. Based on the earlier analysis, the parameters for optimizing the slurry were controlled to a specific gravity of 1.14–1.18 g/cm^3^ and a viscosity of 25–30 s. The following three slurry formulation options were given:

(1) Water–clay–bentonite–CMC-Na–carbonate = 800:110:90:2:0.5
(2) Water–clay–bentonite–CMC-Na–carbonate = 700:110:90:1.5:0.5
(3) Water–clay–bentonite–polyacrylic acid sodium–carbonate = 800:110:90:1:0.5

Among them, Formulation (1) had a specific gravity of 1.14 g/cm^3^ and a viscosity of 29.58 s; Formulation (2) had a specific gravity of 1.17 g/cm^3^ and a viscosity of 28.74 s; and Formulation (3) had a specific gravity of 1.14 g/cm^3^ and a viscosity of 27.31 s. Compared to Formulation (3), Formulations (1) and (2) resulted in a thicker clay film when using CMC-Na as the slurry additive, as this has smaller slurry particles that experience less mechanical wear from agitation. These slurry particles penetrate the formation with greater ease. Formulation (3) has a flocculated structure in the slurry, effectively reducing the water leakage into the formation and hindering the settling of drilling residue. Given the small difference in contribution between the clay and bentonite used in the slurry test for the specific gravity and viscosity, an equal amount of bentonite can replace the clay in order to reduce the sand content of the slurry.

To further verify the interaction between the obtained slurry formulation and the soil, and to study the effectiveness and influencing factors of the slurry wall, the next section employs the PFC3D 6.0 numerical software [39,40,41] to establish a slurry–soil interaction model for slurry-based wall construction in marine sand-containing layers and establish a connection between the slurry parameters, soil parameters, and macro-scale collapse.

## 5. Establishment of Calculation Model and Parameter Testing

### 5.1. Slurry Discrete Element Model

#### 5.1.1. Slurry Discrete Element Model

To ensure the smooth penetration of fine particles during the grouting process, rigid frictionless particles with diameters much smaller than those of soil particles were used to simulate the slurry particles. Referring to previous studies on particle sizes in cement mortar grouting, particles with a radius from 0.5 mm to 0.7 mm were used to simulate the slurry [22]. Based on Basf’s research [42], the density of the slurry particles was set at 1800–2200 kg/m^3^ to simulate the slurry, with a macroscopic density ranging from 1000 to 1200 kg/m^3^. The contact between slurry particles was modeled using a linear elastic contact model. To better simulate the mechanical behavior of fluid materials in terms of discrete elements, the tangential friction coefficient was set to 0, the normal damping ratio was set to 0.2, and the contact parameters of slurry particles are shown in Table 7.

#### 5.1.2. Slurry Viscosity Test Simulation

**Figure 11:**
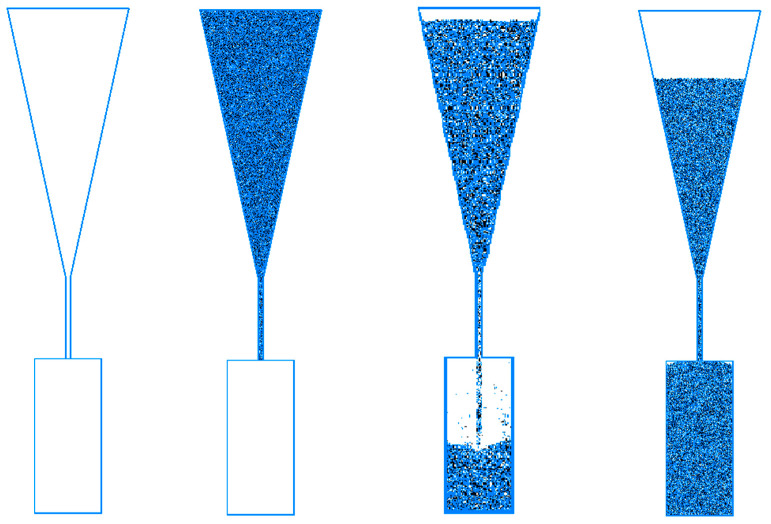
Simulation model of Marsh funnel viscosity test.

To determine the macroscopic viscosity values under the different microscopic parameters of the slurry, a two-dimensional simulation of the Marsh funnel viscosity test was conducted. Using PFC2D, the wall structure shown in Figure 11 was established in order to model the actual size of the Marsh funnel. The wall and small balls were set to be frictionless, and small balls with the particle parameters of the slurry described earlier were used to fill the funnel. A total of 14,330 balls were generated, and the structure was simulated 2000 times to achieve a self-weight balance. Afterward, the wall structure near the spout was removed to simulate the time it took for small balls to fill the lower cylindrical body under the influence of self-weight.

**Figure 12:**
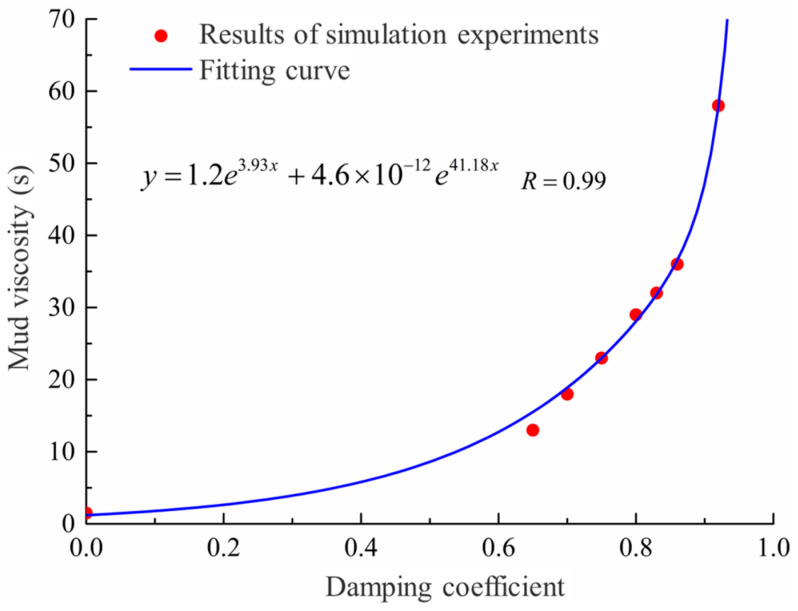
Influence of the local damping coefficient of mud particles on viscosity.

The effects of the micro-scale parameters of each model on the simulation results of mud viscosity are as follows:

1. Mud-particle density has almost no influence on the falling time of the small ball.
2. The ratio of the stiffness of mud particles in contact with each other to the stiffness of the wall has a significant impact on the falling speed of the small ball when the wall is removed. The larger the ratio of mud-particle stiffness to wall stiffness, the faster the initial falling speed of the small ball. When the wall contact stiffness is too large, the small ball does not fall under its own weight. However, after the small ball stabilizes, the wall stiffness has almost no impact on the falling of the small ball.
3. The larger the viscous damping coefficient of mud particles, the slower the falling of mud particles, but the influence of the viscous damping coefficient on the falling of mud particles is relatively small. The viscous damping coefficient was set to 0.6, resulting in a filling time that was only 0.5 s longer than when it was set to 0.2.
4. The local damping coefficient of mud particles has a significant influence on the filling time of falling. When the local damping coefficient was set to 0, the filling time was only 1.5 s. However, when the local damping coefficient was set to a value between 0.65 s and 0.92 s, the filling time could reach between 13 s and 58 s. The influence of the local damping coefficient of mud particles on viscosity is shown in Figure 12.

Based on the above simulation results, it can be determined that the local damping coefficient of particles is the main micro-scale parameter affecting the mud viscosity. The relationship between the macroscopic viscosity of mud and the local damping coefficient of particles is established through the following fitting Equation (7):

(7) $$y=1.2e^{3.93x}+4.6\times 10^{-16}e^{41.18x}$$

#### 5.1.3. Mud-Particle Fluid Characteristics Test

Although the friction coefficient in the mud-particle model is theoretically set to zero, it is important to note that, when using the particle flow to simulate fluids, the macroscopic friction effect of the material is influenced by coupling effects and clogging effects. As a result, the shear strength of the mud that was simulated by the particle flow model is not actually zero.

**Figure 13:**
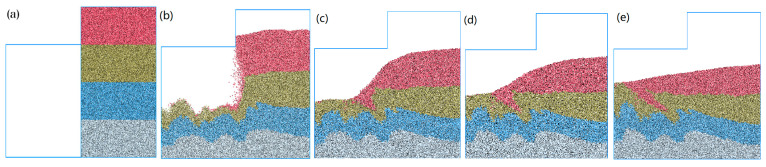
The process of the numerical simulation of the fluid characteristics of mud.

To further evaluate the capabilities of the mud model in terms of accurately capturing viscous fluid behavior, a comprehensive experiment was conducted to analyze the characteristics of mud-particle fluid flow. The experimental results are presented in Figure 13. From Figure 13a to Figure 13e, the bottom mud diffuses first. Over time, this is followed by the higher layers of muck. During the diffusion process, an interface forms between several layers of mud. The contact is irregular due to mud invasion and the influence of the diffusion process. This demonstrates that the observed evolution of the mud flow aligns with the expected behavior of viscous fluids.

Additionally, the natural repose angle of the mud was measured and recorded under different local damping coefficients, as detailed in Table 8. It is worth noting that, in the case of non-viscous particles, the natural repose angle reflects the internal friction angle of the material [43].

### 5.2. Slurry–Soil Interaction: Discrete Element Model

A geological model was established for the MeiSha Zhuanji Project in Yantian District, Shenzhen, that utilized the geotechnical report provided by the prestigious Shenzhen Survey and Design Institute. The model accurately represented the composition of the site, comprising artificial fill layers, gravel layers, and silty clay layers. These are depicted, respectively, by small green balls, brown balls, and light blue balls within the model. To ensure the accuracy of the simulation and minimize boundary effects, the model was meticulously designed with a width of 8 m. The interface between the walls and the soil was modeled as the linear stiffness contact in order to accurately capture the behavior of the soil layers. A total of 13,999 balls were employed in the model to replicate the complex nature of soil layers. These balls serve as elements within the simulation and effectively represent the soil’s composition and interactions. For each soil layer, specific contact models were adopted to accurately simulate the behavior of soil layers and the interactions between the different soil layers. Detailed information regarding these contact models can be found in Table 9, which shows a comprehensive insight into the parameters used for each soil layer.

The densities of the artificial fill layer, gravel–sand layer, and clay layer in the simulation were set to 3.0 g/cm^3^, 3.5 g/cm^3^ and 3.0 g/cm^3^, respectively. In order to simulate the injection of mud, a wall that represented the casing during the mud injection was created at the drill hole position with a spacing of 0.4 m and a depth of 1.5 m. The soil particles at the drill hole position were replaced with purple mud particles in the form of small spheres, and pressure was applied to the wall of the mud particles to simulate different mud pressures within the hole. The interaction between the mud and soil layers was modeled using a linear contact model, the specific parameters of which are outlined in Table 9.

**Figure 14:**
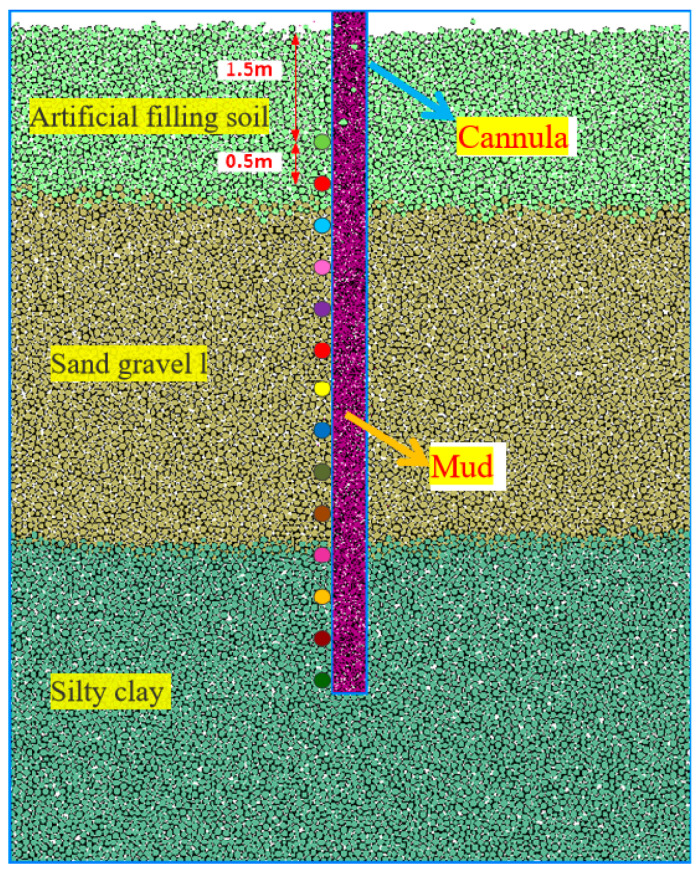
Discrete element model of slurry–soil interaction and arrangement of hole-wall monitoring points.

To effectively monitor the data, 14 monitoring points were strategically placed at the soil positions on the left side of the borehole, going from top to bottom. The first monitoring point, set at a depth of 1.5 m underground, omitted to monitor the deformation of the sleeve part of the pile. The subsequent monitoring points were positioned at intervals of 0.5 m to record the horizontal displacement of the soil. The model and the arrangement of the monitoring points are visualized in Figure 14.

### 5.3. Drill Hole Collapse Phenomenon

**Figure 15:**
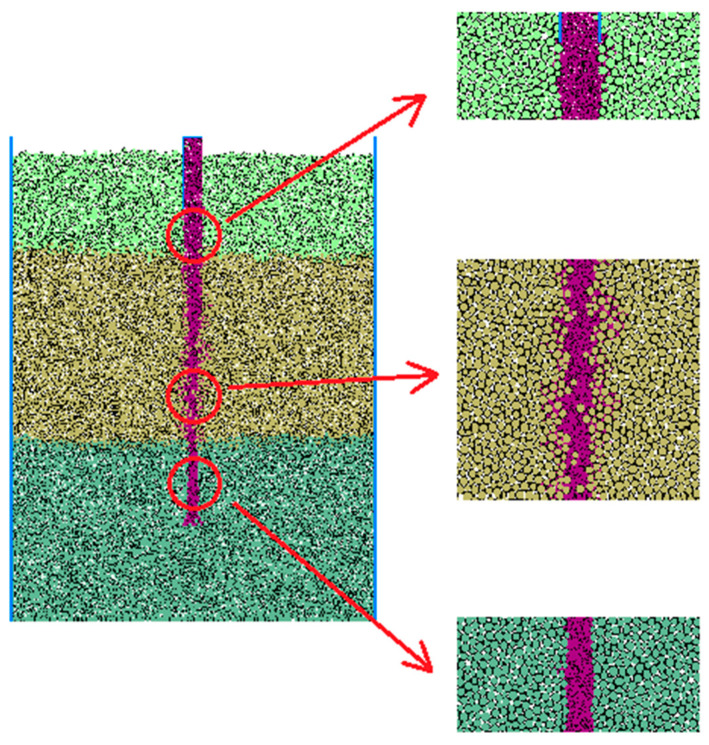
The simulation results of the collapse phenomenon.

For the simulation of collapse, the density of the mud particles used was set as 1.5 g/cm^3^, and the damping coefficient was set as 0.1. No pressure was applied to the mud particles to simulate a situation with low self-weight and low-viscosity mud walls. The stable simulation results obtained from the PFC are presented in Figure 15. It can be observed that, due to the cohesive forces between the particles, there was minimal invasion of mud particles into the artificial fill layer and the clay layer. The stability of the mud wall was enhanced in the artificial fill layer due to its lower gravity in the horizontal direction and the presence of the casing wall. In the clay layer, where the horizontal gravity was higher and the support from the mud was limited, some degree of collapse occurred, although the overall stability of the wall remained satisfactory. In contrast, the gravel–sand layer experienced a significant invasion of mud particles due to the lack of cohesion between the particles, leading to a decline in particle strength within the invaded gravel–sand layer. As a result, the mud particles infiltrated into the hole and caused sliding, resulting in the observed collapse phenomenon.

**Figure 16:**
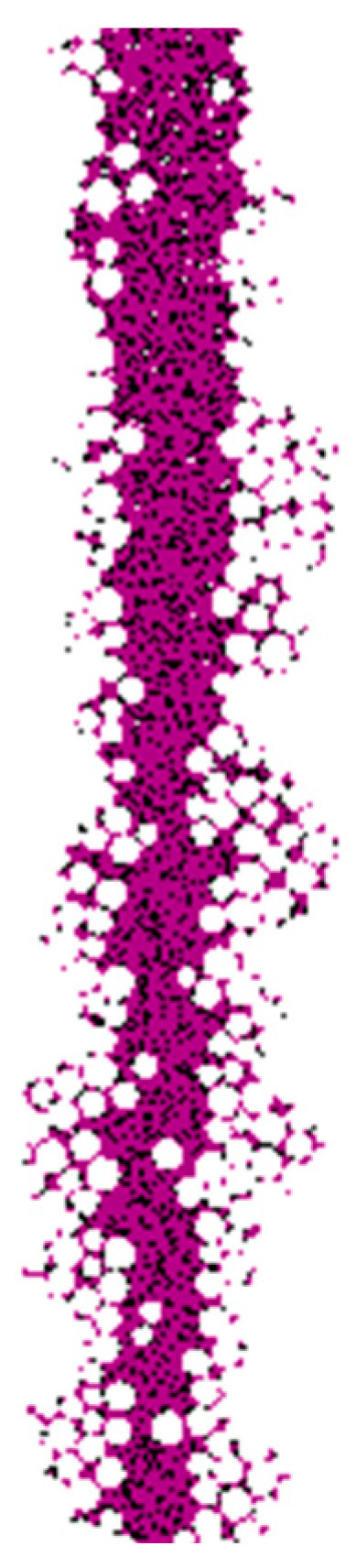
The invasion of mud into the gravel–sand layer.

Figure 16 provides a visual representation of the invasion of mud particles into the gravel–sand layer. In the upper part of the gravel–sand layer, mud-particle invasion is minimal and confined to a small area. However, as the depth increases, a significant invasion of mud particles occurs, primarily on the right side, taking on a V-shaped pattern. The invaded section of the gravel–sand particles maintains stability. At deeper locations, substantial invasion is observed on the left side, with the depth of invasion increasing accordingly. On the right side, particles in the gravel–sand layer start to slide and are transferred into the hole, resulting in the continuous influx of soil into the hole.

**Figure 17:**
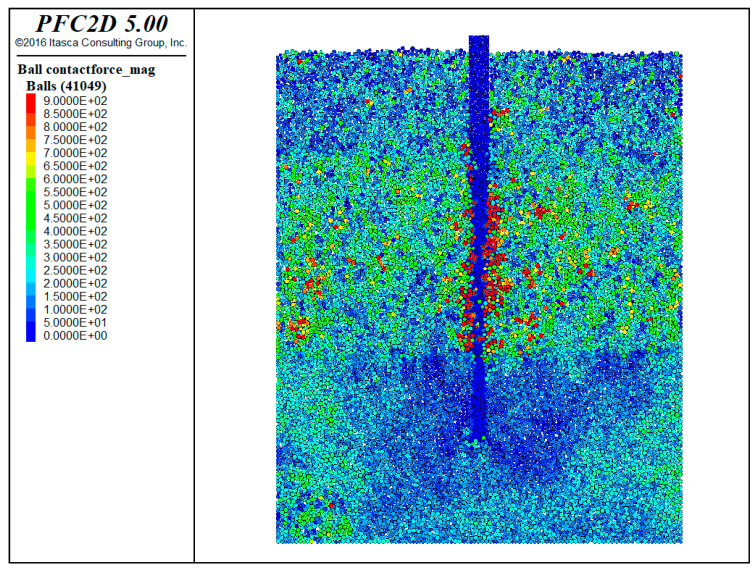
Distribution of contact forces between particles in the collapse simulation.

The distribution of contact forces between soil layers in the collapse simulation can be observed in Figure 17. The contact forces are found to be smaller between the artificial fill layer and the clay layer, while they are larger in the case of the gravel–sand layer. As the mud invades the gravel–sand layer, the contact between soil particles becomes disrupted, resulting in stress concentrations and corresponding collapse areas within the model.

## 6. Simulation Analysis of Mud Wall

### 6.1. Influence of Mud Viscosity

**Figure 18:**
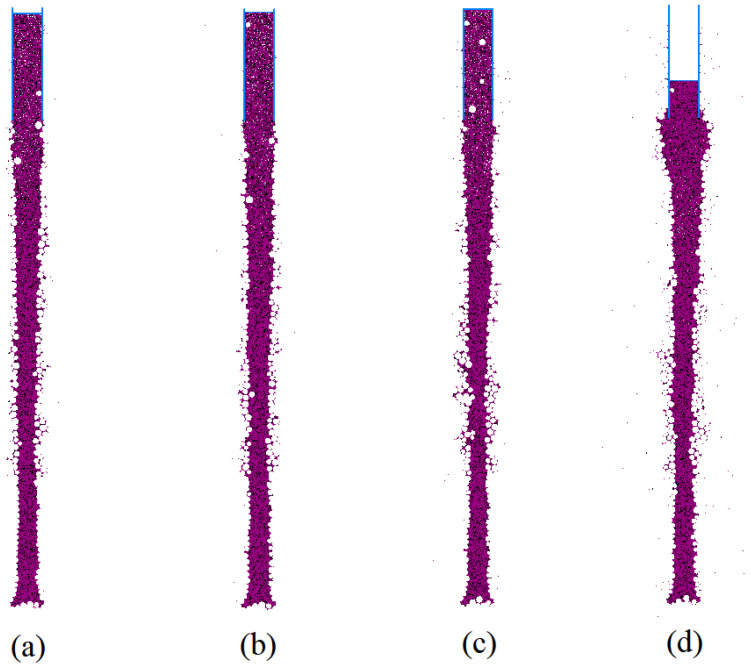
Distribution of mud column under different viscosities local damping: (**a**) 0.9; (**b**) 0.8; (**c**) 0.7; and (**d**) 0.3.

Under a mud-particle density of 1.8 g/cm^3^ and a mud pressure of 100 kPa inside the hole, simulations were performed to compare the effects of different viscosities. The results, which are presented in Figure 18, show the influence of different local damping coefficients (a: 0.9, b: 0.8, c: 0.7, d: 0.3), corresponding to viscosities of 47 s, 28 s, 19 s, and 4 s, respectively, as calculated using Equation (7).

The simulation results indicated that mud penetration mainly occurred in the lower part of the gravel–sand layer. Higher viscosity leads to less mud penetration, and areas with greater mud penetration usually correspond to the severe narrowing of the aperture. When the local damping coefficients are 0.8 or 0.9, the viscosity of the mud typically provides sufficient stability to enable mud-wall protection in the gravel–sand layer. In Figure 18d, it can be observed that the mud pressure inside the hole has a significant impact on hole-wall stability when the viscosity is relatively low. Mud particles are more prone to motion, causing accumulation in the artificial fill layer. This suggests that the appropriate mud pressure inside the hole is closely related to the viscosity of the mud.

**Figure 19:**
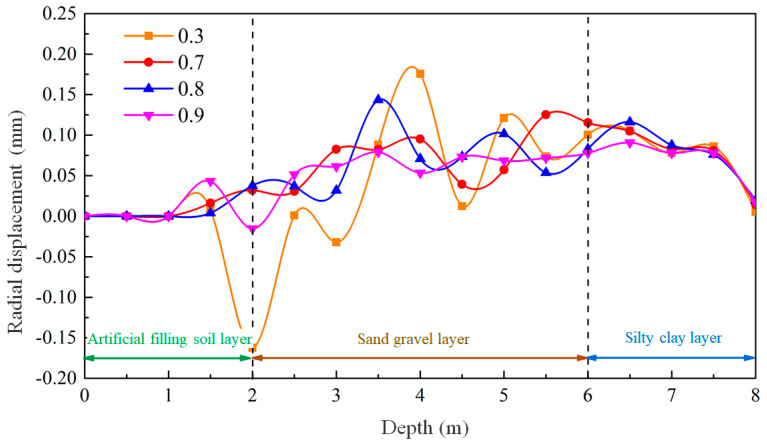
Deformation patterns of the hole wall in different mud-particle-damping conditions.

To further analyze the behavior of the mud wall, the radial displacement of the hole wall is monitored under different mud-particle-damping conditions, as depicted in Figure 19. Positive values correspond to displacement towards the inside of the hole, while negative values represent displacement towards the outside of the hole. As observed, the radial displacement of the hole wall under different viscosities follows a consistent pattern. In the depth range of 0 to 1.5 m, where casing protection is in place, no radial displacement of the hole wall is observed. In the fill layer, between depths of 1.5 and 2 m, a small amount of radial displacement occurs, particularly when the local damping coefficient is 0.3. The depth of this section is relatively small, resulting in a low level of lateral stress being generated by the effect of gravity on the soil. The mud wall provides sufficient support, ensuring the good stability of the hole wall. From a depth of 2 to 6 m, significant radial displacement is observed in the middle section of the gravel–sand layer. The displacement fluctuates greatly with depth, causing the irregular deformation of the hole wall. The relative movement and deformation of the soil particles further disrupt the soil structure, intensifying the invasion of mud particles and resulting in stress concentration. Consequently, the stability of the hole wall decreases, ultimately leading to collapse. In the clay layer at depths of 6 to 8 m, a significant radial displacement is also observed, but the displacement within this section is relatively uniform, with minimal variation with depth. This indicates that, even with an insufficient mud-wall-supporting force, the mud can still effectively exert a uniform force on the hole wall, causing macroscopic shrinkage.

From the perspective of mud-particle damping and the influence of mud viscosity, it is evident that an increase in mud viscosity leads to a decrease in the radial displacement of the hole wall within the gravel–sand layer. This decrease in non-uniform radial displacement gradually transforms into a more uniform displacement, thereby enhancing the stability of the hole wall. Conversely, the effect of increased mud viscosity on the clay layer is limited, resulting in only minor reductions in aperture deformations.

### 6.2. Influence of Mud Specific Gravity

**Figure 20:**
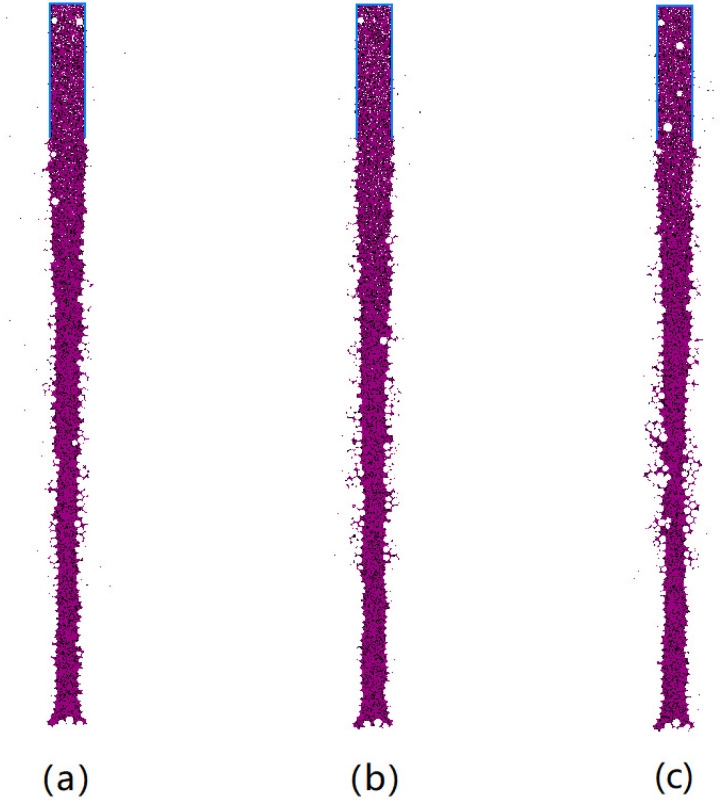
The distribution of mud columns under different specific gravities with mud-particle densities of (**a**) 2.2 g/cm^3^; (**b**) 2.0 g/cm^3^ and (**c**) 1.8 g/cm^3^.

Under a local damping coefficient of 0.7, corresponding to a viscosity of 19 s and a mud pressure of 100 kPa inside the hole, numerical simulation experiments were conducted to investigate the influence of different particle densities on the specific gravity of mud. The results are shown in Figure 20. Figure 20a–c correspond to the mud-particle densities of 2.2 g/cm^3^, 2.0 g/cm^3^, and 1.8 g/cm^3^, respectively, resulting in the mud having specific gravities of 1.2, 1.1, and 1.0.

The simulation results clearly indicate that, under identical conditions, a higher mud density reduces the likelihood of mud infiltration. However, increasing the specific gravity of mud only has a limited contribution to preventing collapse within the gravel–sand layer. The slight increase in the self-weight of the mud, compared to the action of soil particles, does not significantly affect the stability of the mud wall. It is important to note that the amount of mud infiltration exerts a greater influence on the stability of the mud wall.

**Figure 21:**
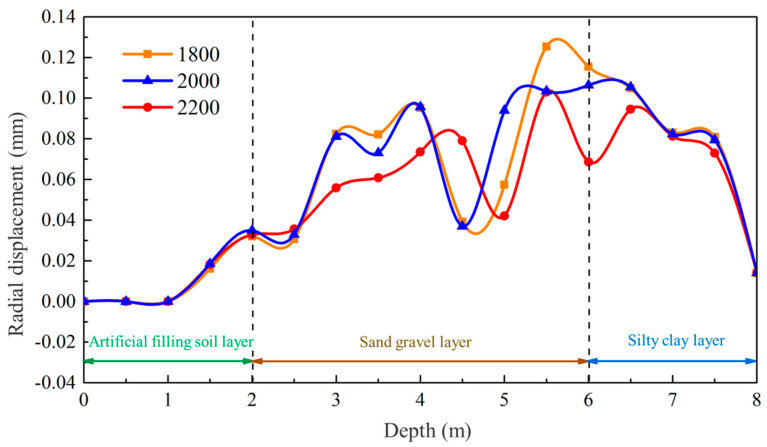
The deformation patterns of the hole wall under different mud-particle densities.

The results of monitoring the radial displacement of the hole wall at different mud-particle densities are shown in Figure 21. The overall radial displacement pattern of the hole wall follows the same pattern observed previously as the depth changes. The increase in mud-particle density or the specific gravity of mud leads to a significant decrease in radial displacement of the hole wall. However, the radial displacement pattern does not change significantly at the point of maximum radial displacement.

### 6.3. Influence of Mud Pressure inside the Hole

**Figure 22:**
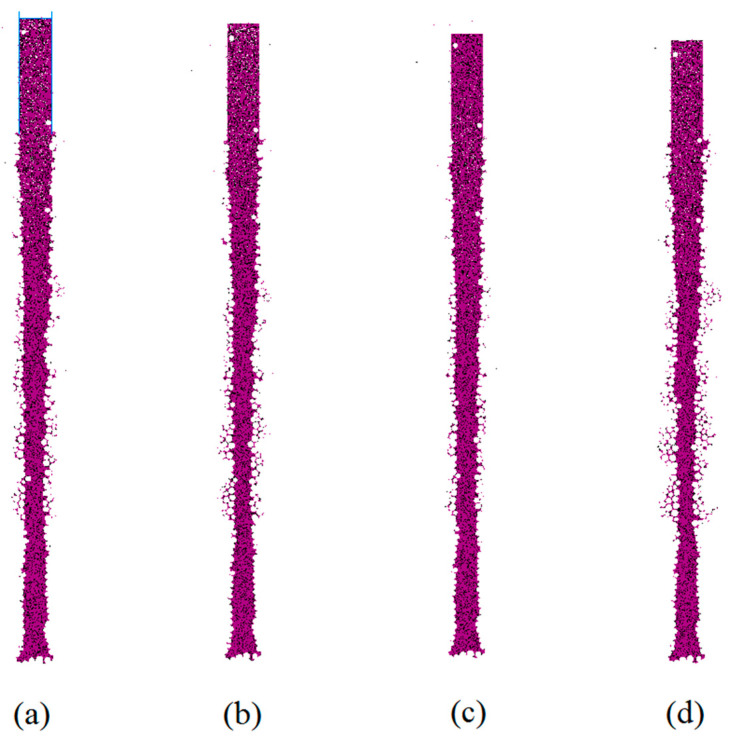
The distribution of the mud column under different mud pressures inside the hole with mud pressures inside the hole of (**a**) 100 kPa; (**b**) 300 kPa; (**c**) 600 kPa; and (**d**) 1000 kPa.

To study the impact of various mud pressures inside the hole on mud-wall behavior, numerical simulation experiments were conducted. The experiments focused on a mud-particle density of 2.2 g/cm^3^, a local damping coefficient of 0.9, a specific gravity of 1.2, and a viscosity of 47 s. Different pressures were applied to the top of the mud column within the hole, and the simulation results were analyzed as depicted in Figure 22.

Figure 22a represents a mud pressure of 100 kPa, where the low mud pressure fails to counterbalance the pressure exerted by the soil. Consequently, the mud column contracts within the gravel–sand layer, and slight infiltration of mud particles is observed. Figure 22b,c correspond to mud pressures of 300 kPa and 600 kPa, respectively. These moderate mud pressures establish a favorable equilibrium between mud forces and gravity within the soil. As a result, the aperture remains adequately maintained, with virtually no infiltration of mud particles.

In contrast, Figure 22d portrays a mud pressure of 1000 kPa, reflecting an excessively high pressure inside the hole. This high mud pressure substantially enhances the dispersion of mud particles within the gravel–sand layer. Consequently, the strength of the penetrated gravel–sand layer diminishes, and the soil undergoes inward movement under the influence of gravity. The compression of the mud column ensues, resulting in a compromised efficacy in terms of protecting the mud wall.

**Figure 23:**
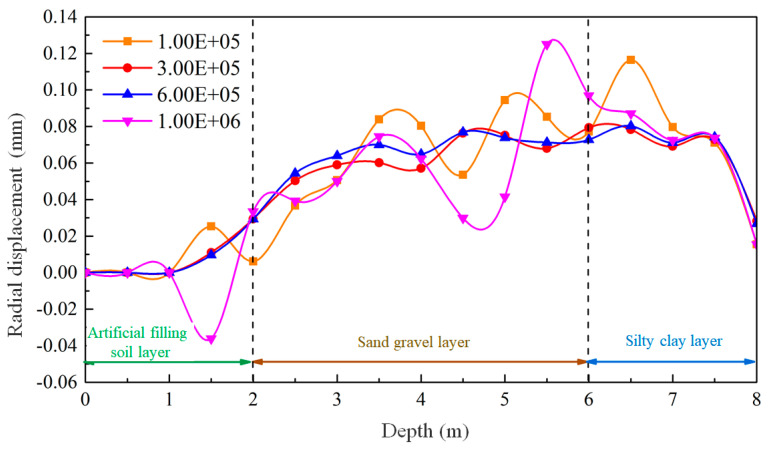
The deformation patterns of the hole wall under different mud pressures inside the hole.

Figure 23 shows the monitoring results of the radial displacement of the hole wall under varying mud pressures within the hole. Consistent with previous observations, the radial displacement pattern aligns with expectations. Specifically, the significant displacement and fluctuation of the hole wall are observed at mud pressures of 100 kPa and 1000 kPa, which is detrimental to the stability of the hole wall. Conversely, when the mud pressures inside the hole are set to 300 kPa and 600 kPa, the displacement and deformation of the hole wall are relatively smaller.

The results indicate that for, the formation of a stable mud wall in the gravel–sand layer, the mud pressure inside the hole should be carefully regulated. Inadequately low mud pressure, relying solely on the self-weight of the mud, fails to counterbalance the pressure exerted by the deep soil layer. Conversely, excessively high mud pressure can accelerate the diffusion of mud particles within the gravel–sand layer, yielding unfavorable outcomes. The appropriate mud pressure inside the hole depends on the mud viscosity and the interaction forces between the mud and the soil layer. Determining an optimal value in practical applications requires the careful consideration of engineering conditions and informed decision making based on experience.

### 6.4. Influence of Drilling Spacing

**Figure 24:**
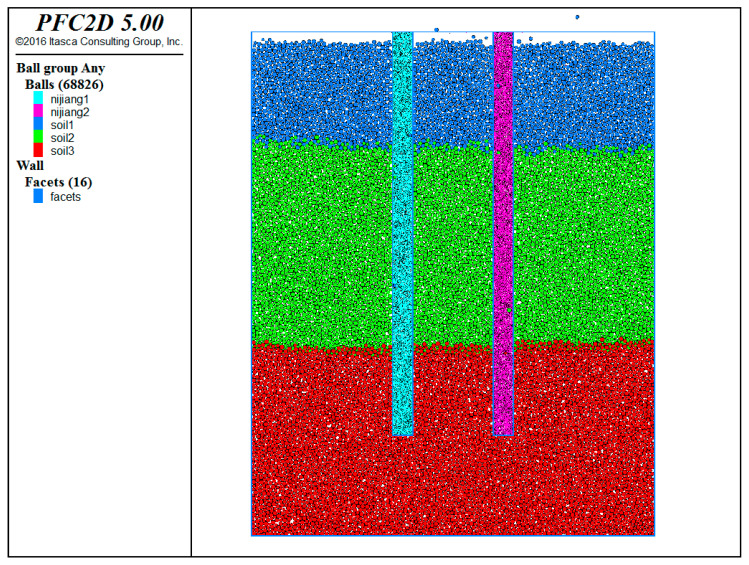
Numerical model on the influence of drilling spacing.

The stability of mud-wall drilling is influenced by the proximity of adjacent drill holes, which necessitates the determination of optimal drilling spacing. In order to investigate the impact of drilling spacing on the effectiveness of mud-wall protection, simulations were conducted on mud-wall piles, with varying spacings between drill holes. A model was created, as depicted in Figure 24, with drill hole center spacings set at 0.8 m, 1.6 m, and 2.0 m. The mud-particle density was fixed at 2.0 g/cm^3^, the damping coefficient was set at 0.7, and the mud pressure inside the hole was set at 100 kPa.

**Figure 25:**
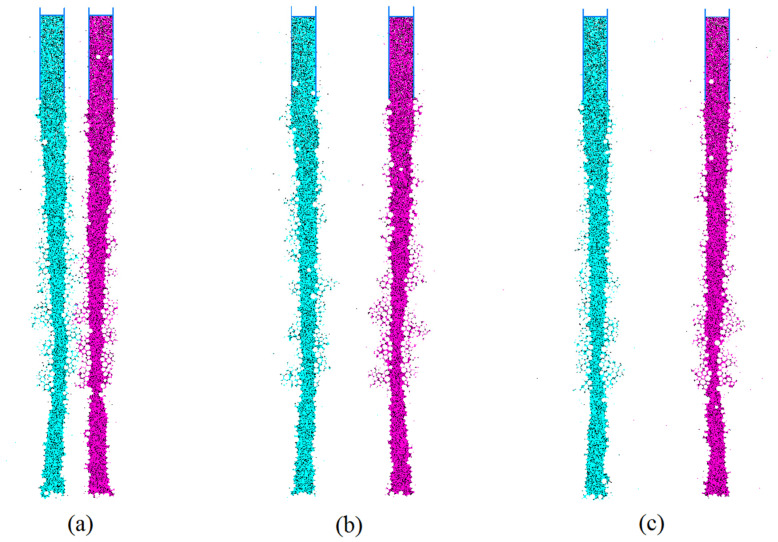
The distribution of the mud column under different drilling spacings of (**a**) 0.8 m; (**b**) 1.6 m; and (**c**) 2.0 m.

The distribution of the mud column under different drilling spacings is presented in Figure 25. The results reveal that, when the drill hole center spacing is 0.8 m, the deformation of adjacent mud-wall piles occurs. This deformation manifests in the form of displacement towards the center of the two drill holes, damage to the wall, and the significant infiltration of mud particles into the surrounding soil. However, as the drilling spacing increases, the displacement is gradually reduced, ultimately becoming negligible when the drill hole spacing reaches 2.0 m.

**Figure 26:**
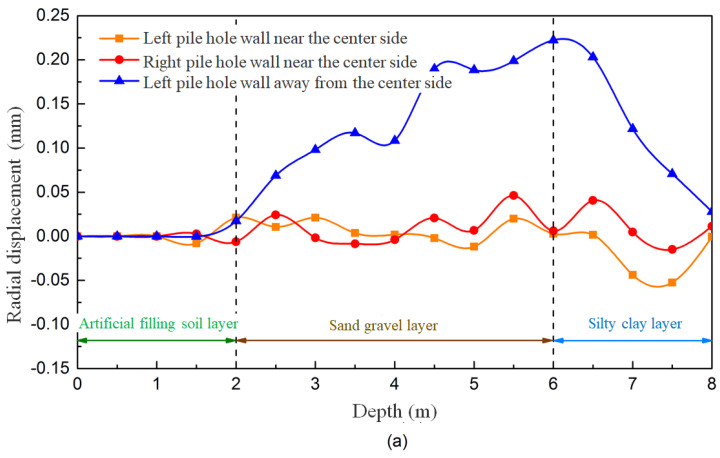

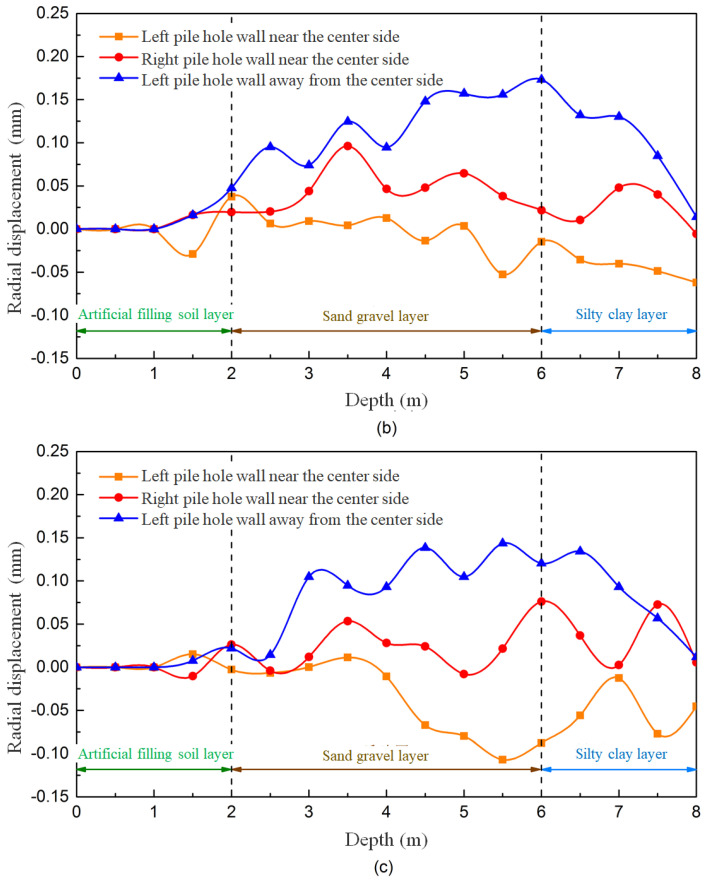
The deformation patterns of the hole wall under different drilling spacings of (**a**) 0.8 m; (**b**) 1.6 m; and (**c**) 2.0 m.

A series of 14 monitoring points was strategically placed on the left hole wall near the center, the left hole wall far from the center, the right hole wall near the center, and the right hole wall far from the center. These monitoring points were positioned at depths that were consistent with the previous sections. The resulting monitoring data are presented in Figure 26. The results reveal that the radial displacement caused by adjacent holes exhibits a relatively consistent pattern, which is primarily concentrated in the deeper sections of the drill holes. Within the same drill hole, greater deformations are observed towards the far center, while relatively smaller deformations occur towards the near center. However, the differences in radial deformation are not significant when the drilling spacing varies. As the drilling spacing increases, the radial displacement of the hole wall towards the far center gradually approaches the near center. This trend continues until a drilling spacing of 2.0 m is reached, at which point the radial displacements on both sides become nearly identical.

## 7. Field Test Result and Discussion

### 7.1. Field Test of the Optimal Ratio of Chemical Mud

**Figure 27:**
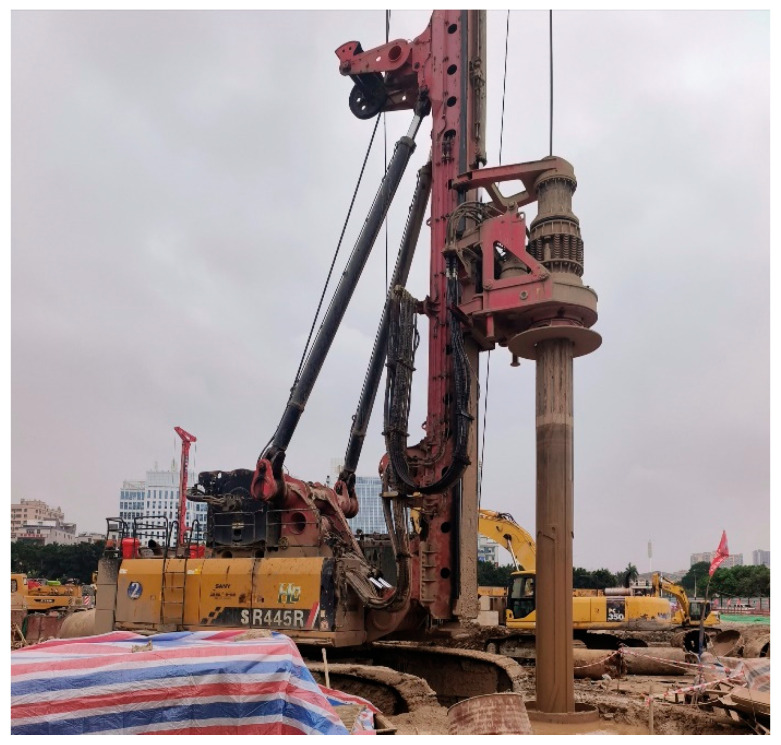
Site construction drawing.

In order to further evaluate the optimal slurry ratio, the bored piles in the areas similar to marine strata were selected for use in the field test. In this area, the collapse holes in the piles became frequent, resulting in the slow progress of the project and large economic losses. In order to compare the chemical mud with ordinary mud, in the process of bored pile drilling, CMC-Na was not added in the early stage until the excavation of the bored sand layer was completed, and the hole collapse phenomenon occurred when the excavation reached the 9.2 m gravel layer. Working according to the geological parameters of the cast-in-place pile, the specific gravity and viscosity requirements of the mud were calculated. In order to prevent the hole collapse phenomenon, the mud was added in time according to the pre-proportioned ratio of water–clay–bentonite–CMC-Na–sodium carbonate = 700:110:90:1.5:0.5 (that is, the mass percentage of each material is 100% of water, 15.7% of clay, 12.8% of bentonite, 0.21% of CMC-Na, 0.07% of sodium carbonate). Chemical mud such as CMC-Na was added to prepare chemical mud for use as bored pile protection mud, and the ratio of the remaining mud materials was controlled according to the recommended ratio. The construction drawing of the control site is shown in the Figure 27. The drilling process and various ratio additions are shown in Table 10.

After adding CMC-Na, the phenomenon of hole collapse does not occur after the bored pile is drilled, and the sediment thickness at the bottom of the hole is reduced from 16 cm to 12 cm, and further reduced to 8 cm. That is to say, adding chemical mud to obtain the recommended optimal ratio can effectively prevent hole collapse and reduce the amount of sediment at the bottom of the hole, that is, the recommended optimal mud ratio can effectively prevent the collapse of bored piles in marine strata.

### 7.2. Discussion on Action Mechanism of Chemical Mud

Based on the results of laboratory ratio tests and field tests, it has been established that the addition of the chemical additive CMC-Na effectively enhances the performance of slurry wall protection. This addition not only prevents the collapse of bored piles, but also reduces the sedimentation at the bottom of the hole. The analysis of numerical simulation results indicates that these can primarily be attributed to the addition of chemical mud. On one hand, it enhances the viscosity of the mud and facilitates its diffusion into the gravel layer. On the other hand, when the bulk density of the slurry counteracts the lateral pressure of the soil layer, it functions as a protective mud wall. However, due to the loose characteristics of the gravel layer and the disturbance effect seen during drilling excavation, the gravel layer becomes even looser, leading to hole collapse. In this process, CMC-Na chemical mud can effectively leverage its interaction with the soil layer effect compared to other ordinary mud, thereby preventing the collapse of the gravel layer in the surrounding rock.

Regarding the interaction with the soil layer of chemical additives, according to the research analysis and Ohama modification mechanism [7,44,45], a large number of cations, e.g., Na^+^, are mixed into the mud after the additive is added. These cations are neutralized, with some negative charges observed on the surface of the slurry, and various contact particles are flocculated into aggregates, thus forming a film on the surface of the hydrated bentonite. Further, this further forms an interpenetrating network structure with the hydration products of bentonite. When the shrinkage of bentonite occurs, the deformation of the newly generated thin film absorbs energy and releases stress. Moreover, when the development of the crack meets the interpenetrating network, the entire additive indicates the formation of microfibers, thereby terminating the development of the crack. Therefore, the additives can change the pore structure of bentonite and improve the adhesion between bentonite slurry and aggregate, resulting in the improvement of the physical and mechanical properties of the slurry.

When considering the role of chemical additives, this paper solely examines their mechanical mechanisms at a macro level, which limits its ability to fully elucidate the wall protection mechanism seen in marine gravel layers. To address this, it is essential to utilize PEC3D numerical simulation software to construct a meso-scale model of chemical mud in the process of borehole wall protection. This will enable a deeper exploration of the mechanical properties of mud under varying chemical mud ratios.

Moreover, conducting micro-scale research [46] to investigate the microstructure of chemical mud under different chemical additive contents and its interaction with the surface of the gravel layer using advanced observation methods is crucial. This approach will provide insights into the mechanisms of action of chemical additives. Therefore, integrating macro–meso–micro perspectives will facilitate a comprehensive understanding of the mechanisms behind chemical mud.

## 8. Guidelines for Engineering Practice

(1) To safeguard slurry walls, use high-viscosity mud to stabilize the sand-layer hole wall. This high-viscosity mud can significantly increase the mud force, eliminating the uneven deformation of the hole wall and limiting mud penetration. It is vital to remember that higher-viscosity mud can improve project performance, but it also increases building complexity. As a result, it is critical to develop a suitable viscosity range throughout project implementation in order to properly exploit the mud’s viscosity properties.
(2) The proportion of mud has a direct relationship with sand layer collapse and clay layer contraction. These problems are most noticeable in the middle and lower sections of the borehole. Increasing the proportion of mud can effectively reduce the deformation of the borehole wall and enhance its stability.
(3) Numerical simulations have indicated that the mud pressure within the hole must be carefully regulated. When generating a mud wall in the gravel–sand layer, excessively low mud pressure in the hole cannot adequately balance the deep soil pressure, relying solely on the self-weight of the mud. Conversely, excessively high mud pressure in the hole can promote the diffusion of mud particles in the gravel–sand layer, leading to unfavorable conditions.
(4) During drilling construction, it is crucial to establish reasonable drilling spacing based on the actual situation. Additionally, the construction organization arrangement of the slurry-retaining wall-drilling pile should be planned in accordance with the spacing to prevent deviations in the drilling position, caused by large drilling spacing.
(5) The addition of CMC-Na to the mud enables effective control of the collapse of cast-in-place piles in marine sand-bearing formations. This treatment method addresses hole collapse without altering the original construction technology. Furthermore, the low cost of CMC-Na yields significant economic benefits, making it a viable solution for widespread implementation in projects.

## 9. Conclusions

Based on the Meisha pile foundation project in Shenzhen, China, this work first investigated a theoretical model of mud acting on borehole wall stability and developed an appropriate theoretical calculation model. Then, studies on mud proportioning and mud characteristics were carried out to investigate the composition and content of mud components, as well as to examine the properties of mud at various proportions. Finally, a numerical investigation of borehole stability under mud-wall protection was carried out using discrete element methods, including the modeling of the mud’s viscous-fluid particle flow and an analysis of mud-particle characteristics. The following conclusions may be drawn:

(1) Based on the theory of circular borehole expansion, the borehole wall’s stability is determined using two parameters, which are mud slurry stability and mud-film stability. These define the safety factor for borehole wall stability under the influence of mud slurry. An analysis based on engineering practices examines the influencing factors on borehole wall stability and outlines the characteristics of borehole instability in varying geological conditions.
(2) Elevating the content of solid-phase components (such as clay and bentonite) notably increases the mud slurry’s density. With increasing water content, the density, viscosity and sand content of the mud slurry decrease. Additives like sodium carboxymethyl cellulose and sodium polyacrylate primarily boost mud slurry viscosity. Sodium polyacrylate also exhibits significant flocculation properties, whereas barium sulfate primarily enhances the density. Three optimized schemes for mud slurry proportion are proposed based on similar construction cases.
(3) The created mud slurry fluid model adequately simulates density, viscosity, and other fluid parameters. In the gravel–sand layers of marine strata, the significant incursion of mud slurry particles produces uneven borehole wall deformation, which ultimately leads to hole collapse. In contrast, in the powdery clay layers, restricted mud slurry invasion causes consistent borehole wall deformation, resulting in hole shrinkage.
(4) Increased mud slurry viscosity minimizes radial displacement in borehole walls inside gravel–sand layers. As viscosity increases, radial displacement becomes more uniform. However, increasing viscosity increases building difficulties, underlining the importance of determining an ideal viscosity range in practical engineering.
(5) Maintaining proper mud slurry pressure and spacing is crucial during drilling operations. The inadequate mud slurry pressure, which is exclusively dependent on the mud slurry weight, fails to balance the deep soil pressure. Increased mud slurry density greatly lowers drill-wall radial displacement. Excessive mud slurry pressure causes mud slurry particles to diffuse in gravel–sand layers, resulting in undesirable conditions. Furthermore, when the drilling spacing is too tiny, the hole deviates towards the center point, whereas when it is too large, the drilling position deviates.
(6) Through the on-site chemical mud-wall-protection experiment of bored piles in a sand layer, it is concluded that CMC-Na chemical mud can effectively prevent the collapse of the hole and reduce the sand content at the bottom of the hole.

## Figures and Tables

**Table 1:** Recommended rock–soil parameters for supporting excavation-pit design in the site report.

Formation Name and Genetic Code	Layer Number	Soil State	Weight γ (kN/m^3^)	Consolidation Quick Shear		Critical Bond Strength Standard Value q_sik_ (kPa)	Friction Coefficient μ of Grouted Body
				Internal Friction Angle φ (°)	Cohesion *c* (kPa)		
Artificial fill (Qml)	1	Loose	18.0	14	7	20	0.25
Gravel–sand (Q4mc)	2–1	Loose to slightly dense	20.0	32	/	60	0.4
Silty clay (Q4mc)	2–2	Soft plastic to plastic	16.0	3	6	14	/
Silty clay (Q3al + pl)	3	Plastic to hard plastic	19.0	14	23	60	0.25
Sandy clay (Qel)	4	Plastic to hard plastic	18.6	19	23	80	0.35

**Table 2:** Experimental instrument parameters and purposes.

Instrument Name	Unit, and Precision	Purpose
NB-1 Mud Specific Gravity Meter	g/cm^3^ (0.01 g/cm^3^)	Measure the specific gravity of mud
1006 Mud Viscosity Meter	S (0.01 s)	Measure the viscosity of mud
NA-1 Mud Sand Content Meter	0.01%	Measure the sand content of mud
pH test paper		Measure the pH value of mud
Precision electronic scale	g	Weighing test materials
Microstirrer	r/min	Used for stirring mud
65-mesh sieve	--	Sieve the clay used for testing
Graduated cylinder, several beakers	several	Measure and hold liquid for volume measurement

**Table 3:** Experimental scheme for the effects of different slurry components on mud slurry characteristics.

Reference Number	Water (mL)	Clay (g)	Bentonite (g)	CMC-Na (g)	Sodium Carbonate (g)	Specific Gravity	Viscosity (s)	Sand Content (%)	PH
1	800	0	50	1	0.5	1.04	18.35	0.10	9.5
2	800	20	50	1	0.5	1.05	18.69	0.60	9
3	800	40	50	1	0.5	1.06	20.39	2.25	9.5
4	800	60	50	1	0.5	1.08	20.07	2.50	9.5
5	800	80	50	1	0.5	1.10	21.35	3.50	9.5
6	800	100	50	1	0.5	1.11	20.34	4.50	9
7	800	50	0	1	0.5	1.03	17.43	2.00	9.5
8	800	50	20	1	0.5	1.04	17.87	1.80	9.5
9	800	50	40	1	0.5	1.07	18.54	2.10	9.5
10	800	50	60	1	0.5	1.08	19.36	1.60	8.8
11	800	50	80	1	0.5	1.10	21.04	2.70	8.5
12	800	50	100	1	0.6	1.12	20.47	3.00	9
13	600	110	90	1	0.5	1.21	30.45	12.53	8.5
14	700	110	90	1	0.5	1.17	23.27	9.23	9
15	800	110	90	1	0.5	1.14	22.35	7.57	8.5
16	900	110	90	1	0.5	1.12	21.61	7.32	8.5
17	1000	110	90	1	0.5	1.11	19.98	7.16	8.5
18	1000	110	90	1	0.5	1.11	19.92	7.12	8.5
19	1000	110	90	2	0.5	1.11	26.38	6.53	9
20	1000	110	90	3.5	0.5	1.13	31.04	7.65	9.6
21	1000	110	90	8	0.5	1.13	284.44	7.10	9

**Table 4:** Experimental scheme for the influence of sodium polyacrylate on mud slurry characteristics.

Reference Number	Water (mL)	Clay (g)	Bentonite (g)	Sodium Polyacrylate (g)	Sodium Carbonate (g)	Specific Gravity	Viscosity (s)	Sand Content (%)	PH
22	1000	110	90	1	0.5	1.12	23.96	30.27	9
23	1000	110	90	2	0.5	1.12	34.74	--	9.5
24	1000	110	90	3.5	0.5	1.12	49.71	--	9.1
25	1000	110	90	8	0.5	1.12	346.25	--	9.5

**Table 5:** Experimental scheme for the influence of barium sulfate on mud slurry characteristics.

Reference Number	Water (mL)	Clay (g)	Bentonite (g)	Barium Sulfate (g)	Sodium Carbonate (g)	Specific Gravity	Viscosity (s)	Sand Content (%)	PH
26	1000	110	90	15	0.5	1.13	21.47	7.12	9.2
27	1000	110	90	60	0.5	1.16	30.66	7.37	9.5
28	1000	110	90	100	0.5	1.18	34.41	9.16	9.5
29	1000	110	90	150	0.5	1.21	38.49	11.35	9.5

**Table 6:** Analysis of slurry viscosity in engineering examples.

Project Name	Formation Used	Slurry Materials	Slurry Viscosity
Cambodia Bassac River East Channel Bridge	Fine sand formation	Clay, bentonite, CMC-Na, sodium carbonate, water	18–25 s
Wuhan Erqi Changjiang River Bridge supporting project	Thick sand formation	Bentonite, CMC-Na, sodium carbonate, water	25–28 s
Yu Men Kou Yellow River Highway Bridge project	Saturated liquefied sand formation	Bentonite, polyacrylamide, sodium carbonate, water	26–35 s
Hong Kong-Zhuhai-Macao Bridge Lingdingyang West Beach project	Sand formation	Bentonite, CMC-Na, polyacrylamide, sodium carbonate, water	22 s

**Table 7:** Slurry-particle contact-model parameters.

Model Type	$\mathrm{Normal}\;\mathrm{Stiffness}\;k_{n}$	Tangent Friction Coefficient μ	$\mathrm{Normal}\;\mathrm{Damping}\;\mathrm{Ratio}\;\beta_{n}$	$\mathrm{Tangential}\;\mathrm{Damping}\;\mathrm{Ratio}\;\beta_{s}$
Parameter Value	3.2 × 10^5^	0	0.2	0

**Table 8:** Natural repose angle of mud under different local damping coefficients.

Local Damping Coefficient	0	0.3	0.5	0.6	0.7	0.8	0.9
Repose angle (°)	5	5.6	6.2	6.8	7.5	7.8	8.1

**Table 9:** Contact model parameters for different soil layer.

Soil Layer	Model Type	kn	ks	fric	dp_nratio	pb_kn	pb_ks	pb_ten	pb_coh	pb_rmul	dp_sratio	cb_tenf	cb_shearf
artificial fill	linearcbond	3 × 10^6^	-	0.2	0.2	-	-	-	-	-	0	1 × 10^10^	1 × 10^10^
gravel	linear	5 × 10^6^	-	0.3	0.2	-	-	-	-	-	0	-	-
silty clay	linearpbond	3 × 10^6^	3 × 10^6^	0.2	0.2	1 × 10^8^	1 × 10^8^	1 × 10^6^	1 × 10^6^	0.8	-	-	-
slurry–soil	linear	3 × 10^5^	3 × 10^5^	0.2	0.2	-	-	-	-	-	0	-	-

**Table 10:** Mud preparation during drilling construction.

Drilling Depth/m	0~9	9~18	18~21
Stratigraphy condition	0~2.8 m is silt layer, 2.8~9 m is sand layer, 9~18.05 m is gravel layer, 18.05~21 m is slightly weathered rock layer.		
Water/kg	8124.75	8124.75	2708.25
Bentonite/kg	1039.97	1039.97	346.66
Clay/kg	1275.59	1275.59	425.2
CMC-Na/kg	0	17.06	5.69
Specific gravity	1.2	1.2	1.2
Sediment content	2.5	2.5	2.5
PH	9	9	9
Viscosity/s	31	31.5	31
Sediment thickness at the bottom of the hole/cm	16 cm	12 cm	8 cm
Description of the drill hole	CMC-Na carboxymethylcellulose sodium was not used before the collapse. CMC-Na was added after the 9.2 m collapse, and no collapse occurred after CMC-Na was added.		

## Data Availability

Data are contained within the article.
